# A Deep Learning Analysis Reveals Nitrogen-Doped Graphene Quantum Dots Damage Neurons of Nematode *Caenorhabditis elegans*

**DOI:** 10.3390/nano11123314

**Published:** 2021-12-07

**Authors:** Hongsheng Xu, Xinyu Wang, Xiaomeng Zhang, Jin Cheng, Jixiang Zhang, Min Chen, Tianshu Wu

**Affiliations:** 1 College of Energy and Electrical Engineering, Hohai University, Nanjing 210098, China; hsxu_nj@163.com; 2 Key Laboratory of Environmental Medicine and Engineering, Ministry of Education, School of Public Health, Southeast University, Nanjing 210009, China; wangxinyu_990309@163.com (X.W.); 18852073768@163.com (X.Z.); chengjin1123@163.com (J.C.); 213193743@seu.edu.cn (J.Z.); 220214003@seu.edu.cn (M.C.)

**Keywords:** nanotoxicity, neurotoxicity, neurobehavior, machine learning, phenotyping

## Abstract

Along with the rapidly increasing applications of nitrogen-doped graphene quantum dots (N-GQDs) in the field of biomedicine, the exposure of N-GQDs undoubtedly pose a risk to the health of human beings, especially in the nervous system. In view of the lack of data from in vivo studies, this study used the nematode *Caenorhabditis elegans* (*C. elegans*), which has become a valuable animal model in nanotoxicological studies due to its multiple advantages, to undertake a bio-safety assessment of N-GQDs in the nervous system with the assistance of a deep learning model. The findings suggested that accumulated N-GQDs in the nematodes’ bodies damaged their normal behavior in a dose- and time-dependent manner, and the impairments of the nervous system were obviously severe when the exposure dosages were above 100 μg/mL. When assessing the morphological changes of neurons caused by N-GQDs, a quantitative image-based analysis based on a deep neural network algorithm (YOLACT) was used because traditional image-based analysis is labor-intensive and limited to qualitative evaluation. The quantitative results indicated that N-GQDs damaged dopaminergic and glutamatergic neurons, which are involved in the neurotoxic effects of N-GQDs in the nematode *C. elegans*. This study not only suggests a fast and economic *C. elegans* model to undertake the risk assessment of nanomaterials in the nervous system, but also provides a valuable deep learning approach to quantitatively track subtle morphological changes of neurons at an unbiased level in a nanotoxicological study using *C. elegans*.

## 1. Introduction

Graphene quantum dots (GQDs) are a relatively new type of quantum dots (QDs), which have not only excellent properties of graphene, but also novel characteristics of QDs, including quantum confinement effect and size effect. Therefore, GQDs present significant potentials in the applications of environmental monitoring and pollutant detection due to their excellent properties, such as excellent optical properties, low toxicity, good biocompatibility and low preparation cost, among which optical properties, including strong photoluminescence (PL), fluorescent stability and tunable luminous color are key features, such they can be considered as a new class of fluorophores overcoming weaknesses of organic dyes commonly used as fluorophores but that suffer from photobleaching and do not allow long-term exposure to excitation sources [1,2].

GQDs that contains different functional groups could be useful candidates to be prepared for specific application requiring better stability and fluorescence intensity. Several studies have show that doping GQDs with nitrogen, i.e., nitrogen-doped GQDs (N-GQDs), processes a distinct structure, and improves their activities towards catalysis and appearance of up-conversion photoluminescence in order to meet the demands of potential application in sensors in the environment [3,4]. The doping strategy are beneficial for achieving better performance in the environmental application of GQDs [4]. Along with the increasing applications of N-GQDs, their exposure to the public in the environment undoubtedly pose a threat to human health. Recently, graphene-based nanomaterials have been found to easily reach the central nervous system (CNS) through multiple ways, so their neurotoxic effects get more attention from researchers [5,6]. Unfortunately, the information on the neurotoxicity of N-GQDs is rare.

It is still controversial as to whether GQDs exert toxic effects in living bodies due to their inherent graphene-like properties, because the nanotoxicity of graphene have been extensively assessed during the past decade [7,8,9]. Even though some studies suggested that N-GQDs presented low cytotoxicity and good biocompatibility [10,11], some researchers found the oxidative stress damage is caused by N-GQDs [12,13]. However, current knowledge of low toxicity of GQDs was mainly obtained from in vitro rather than in vivo toxicity assessments. Therefore, it is of great importance to assess the potential health risks of GQDs in living organisms.

Although mice are commonly used animal models in nanotoxicology studies, these are confronted with some weaknesses, such as being time-consuming, high cost and sampling constraints. In present years, an animal model called *C. elegans* have been widely used for biosafety assessment of nanomaterials due to many virtues, including transparent body, high fertility, short life cycle, low husbandry cost and conserved evolutionary modules in the genome [14,15]. This organism has been reported to be used in toxicological studies of different nanomateirals, including carbon-based nanoparticles and quantum dots [14,16]. Meanwhile, the number of applications of *C. elegans* in assessing nanomaterials in the nervous system, especially adverse morphological changes of neurons, is increasing [17,18,19,20]. Therefore, in this study, the neurotoxicity of N-GQDs was assessed in this animal model and we focused on the adverse effects of N-GQDs in different types of neurons.

As we know, assessment on the characterization of neurons is usually limited in the number of beads by manual counting of fluorescent images in neurotoxicity studies using *C. elegans*. This traditional image processing approach typically relies on intensity difference for image segmentation so is unable to perform the challenging segmentation. In addition, quantitative analysis of morphological changes in neurons is challenging and manual inspections to quantify the number of protrusions are time-consuming, involve human bias and are labor intensive. Moreover, manual counting of changes provides limited information and makes thorough analysis of the complex phenotypes acquired in fluorescence images unfeasible. Recently, machine learning and deep phenotyping have been implemented in several studies to quantitatively analyze neurodegeneration morphology of *C. elegans* to detect neurons and describe their patterns [21,22,23]. In this study, with the assistance of a deep neural network algorithm (YOLACT), we found that N-GQDs could impair neurobehaviors in *C. elegans* through damaging dopaminergic neurons and glutamatergic neurons. The quantitative data not only provide additional information about the observed morphological changes, but also enable predicting the toxicity status of a nematode exposed to nanomaterials.

## 2. Results

### 2.1. Nitrogen-Doped Graphene Quantum Dots (N-GQDs) Caused Neurotoxic Effects in Nematode C. elegans

**Figure 1:**
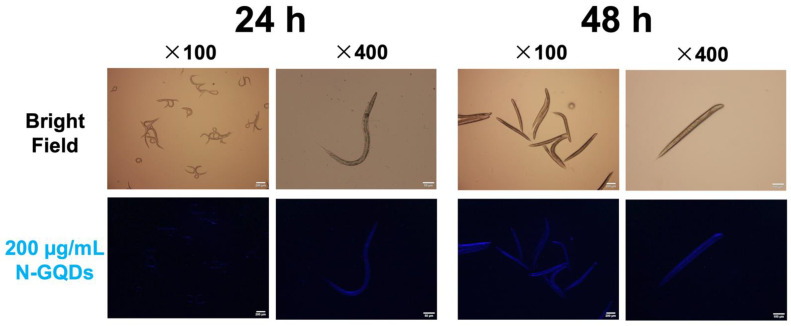
The accumulation of nitrogen-doped graphene quantum dots (N-GQDs) in the nematode *C. elegans*. Representative fluorescent images of nematodes treated with 200 µg/mL N-GQDs for 24 h and 48 h. The N-GQDs showed blue. Scare bars: 200 µm and 50 µm.

Owning to the excellent fluorescence of N-GQDs and the transparent body of nematodes *C. elegans*, the accumulation of N-GQDs in the nematodes body of wild-type N2 *C. elegans* without autofluorescence could be visually observed through a fluorescent microscope. When nematodes were exposed to 200 µg/mL N-GQDs for 24 h, the fluorescence of N-GQDs showing blue, which is the color of spontaneous fluorescence from N-GQDs, could be observed in the bodies, mainly accumulated in the intestinal system, while the fluorescent intensity in nematodes treated with N-GQDs at the same dose for 48 h were stronger than those for 24 h and fluorescence could be observed in the whole body, including the head (Figure 1). It seems that N-GQDs can be devoured by nematodes and distributed into their whole bodies, which could then exert some adverse effects.

**Figure 2:**
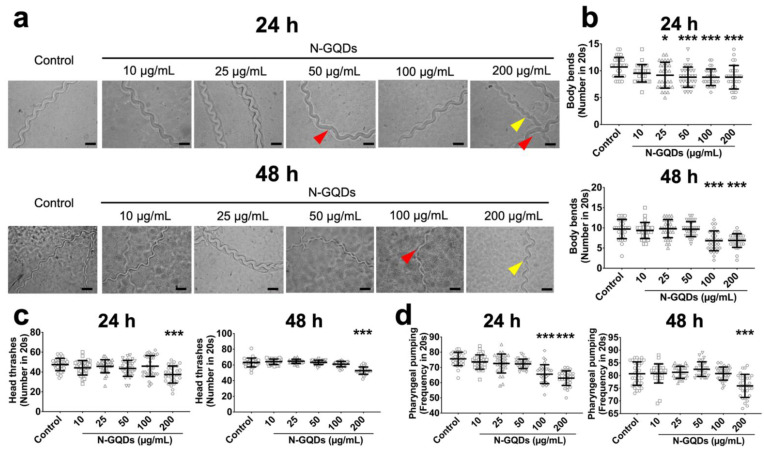
The neurotoxic effects of N-GQDs in wide-type N2 *C. elegans*. (**a**) representative images of the forward locomotion trackers of nematodes. Red arrows indicate disjointed movement, yellow arrow indicate serrated movement. Scale bar: 200 µm; The quantity of body bends (**b**), the quantity of head thrashes (**c**) and the frequency of pharyngeal pumping (**d**) in nematodes. L4-larvae of N2 and L1-larvae of N2 were treated with 0, 10, 25, 50, 100 and 200 µg/mL N-GQDs for 24 h without food and 48 h with food, respectively (*n* = 30). Data are shown as mean + SD of three independent experiments. The one-way analysis of variance (ANOVA) followed by the Dunnett’s *t*-test were used to determine statistical significance (* *p* < 0.05, *** *p* < 0.001 vs. the control).

The locomotion alteration in nematodes were generally accepted by researchers to assess the neurotoxicity of nanomaterials [20,24]. The forward locomotion of *C. elegans* that is controlled by the dorsal and ventral longitudinal bundle muscles and the normal movement trace, also known as locomotion tracker, of forward locomotion is smooth, continuous and sinusoidal [25]. Firstly, the forward locomotion trackers were used to evaluate the neurotoxicity of N-GQDs and representative images suggested that N-GQDs exposure at high doses, including 100 and 200 µg/mL, caused the movement trace of forward locomotion of nematodes to become a little disjointed and similar to serration (Figure 2a). Moreover, N-GQDs exposure at doses over 25 µg/mL for 24 h and over 100 µg/mL for 48 h statistically significantly reduced the number of body bends in nematodes (Figure 2b), while 200 µg/mL N-GQDs exposure for 24 h and 48 h both significantly deceased the quantity of head thrashes (Figure 2c). Furthermore, reductions in the frequency of pharyngeal pumping were observed in nematodes treated with N-GQDs at doses over 100 µg/mL for 24 h and 200 µg/mL for 48 h (Figure 2d). The impairments of body bend, head thrash and pharyngeal pumping indicated the neurotoxic effects of N-GQDs in *C. elegans*.

### 2.2. The Neurotoxicity of N-GQDs Was Associated with Damaging Dopaminergic Neurons and Glutamatergic Neurons in Nematode C. elegans

Behaviors reflect nervous system activity and is dependent on multiple factors, including neuronal structure and function [15,26]. A broadly applied classification of neurons is based on the type of neurotransmitter that a neuron produces, i.e., neurotransmitter phenotype [27]. Five commonly neurotransmitters comprising the *C. elegans* nervous system are dopamine, glutamate, γ-aminobutyric acid, choline and serotonin [28,29]. Here, five genes that are usually used as promoters to derive GFP to identify the five different types of neurons were chosen to assess the toxic targets of N-GQDs (Table S1).

**Figure 3:**
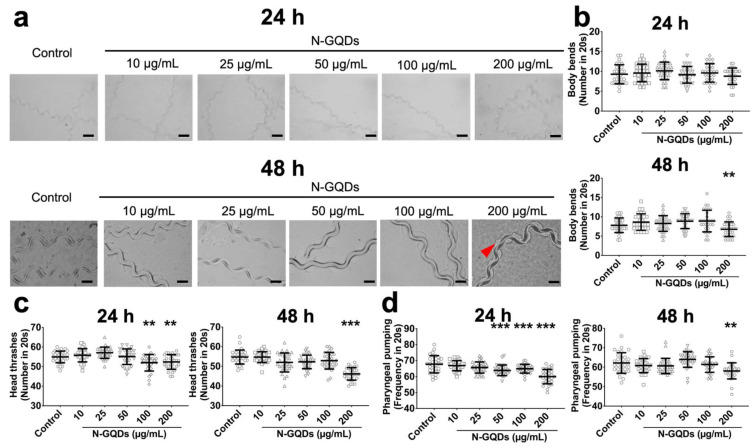
The neurotoxic effects of N-GQDs in transgenic strain of BZ555 *C. elegans*. (**a**) representative images of the forward locomotion trackers of nematodes. Red arrows indicate disjointed movement. Scale bar: 200 µm; The quantity of body bends (**b**), the quantity of head thrashes (**c**) and the frequency of pharyngeal pumping (**d**) in nematodes. L4-larvae of BZ555 and L1-larvae of BZ555 were treated with 0, 10, 25, 50, 100 and 200 µg/mL N-GQDs for 24 h without food and 48 h with food, respectively (*n* = 30). Data are shown as mean + SD of three independent experiments. The one-way ANOVA followed by the Dunnett’s *t*-test were used to determine statistical significance (** *p* < 0.01, *** *p* < 0.001 vs. the control).

**Figure 4:**
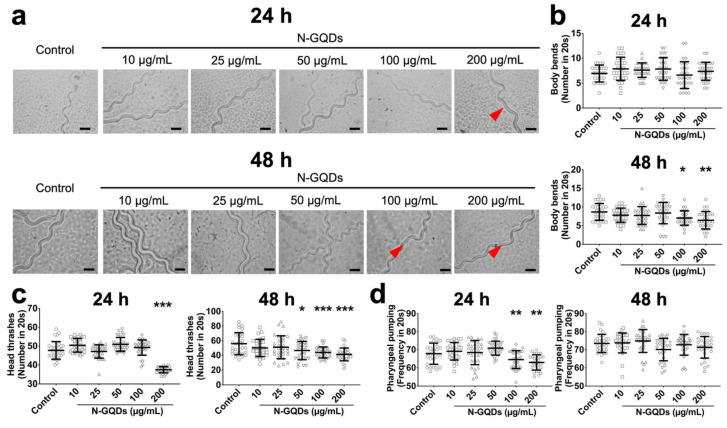
The neurotoxic effects of N-GQDs in transgenic strain of DA1240 *C. elegans*. (**a**) representative images of the forward locomotion trackers of nematodes. Red arrows indicate disjointed movement. Scale bar: 200 µm; The quantity of body bends (**b**), the quantity of head thrashes (**c**) and the frequency of pharyngeal pumping (**d**) in nematodes. L4-larvae of DA1240 and L1-larvae of DA1240 were treated with 0, 10, 25, 50, 100 and 200 µg/mL N-GQDs for 24 h without food and 48 h with food, respectively (*n* = 30). Data are shown as mean + SD of three independent experiments. The one-way ANOVA followed by the Dunnett’s *t*-test were used to determine statistical significance (* *p* < 0.05, ** *p* < 0.01, *** *p* < 0.001 vs. the control).

The results suggested that only expressions of genes dat-1 and eat-4 significantly downregulated in nematodes with 100 and 200 µg/mL N-GQDs treatments for 24 h and 48 h, which indicated that N-GQDs seem to have a greater neurotoxic effect on dopaminergic neurons and glutamatergic neurons than others (Figure S1). Therefore, the *C. elegans* transgenic strains BZ555 and DA1240 were used here, where dopaminergic and glutamatergic neuron-specific proteins were tagged with GFP, to assess the damages caused by N-GQDs on the protein synthesis and neuron structure. When the two transgenic strains were exposed to 10~200 µg/mL N-GQDs for 24 h and 48 h, behavioral deficits in locomotion, including forward locomotion, body bend and head thrash (Figure 3a,c and Figure 4a,c) and pharyngeal pumping (Figure 3d and Figure 4d) were observed to different extents.

### 2.3. The Application of Training YOLACT Algorithm in Image Segmentation and Analysis

**Figure 5:**
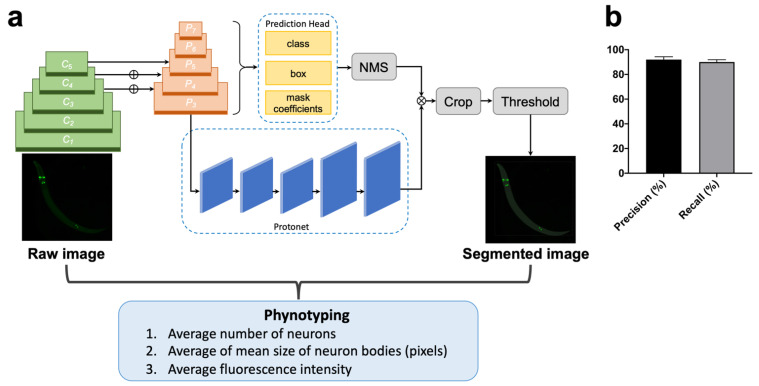
The deep learning analysis identified phenotyping of neurons in *C. elegans*. (**a**) System diagram and network structure of morphological changes of neurons in *C. elegans* treated with N-GQDs based on YOLACT; (**b**) The precision and recall of segmentation to test the performance of the YOLACT algorithm.

We employed the YOLACT model to identify and mask the objects (the target fluorescent neurons), as well as to obtain the fluorescent intensity of objects, and thereby quantitatively analyze N-GQDs-induced morphological changes in neurons. The system diagram and network structure are shown in Figure 5a. The proposed scheme was composed of backbone, FPN, Protonet, and prediction head for instance fluorescent nematode image segmentation. The input to YOLACT is a three-channel fluorescent nematode image, whose default size is 550 × 550 pixels. A residual network (ResNet-101 [30]) combined with FPN was used to extract multiscale feature maps from a raw image, then these extracted feature maps were transmitted into two parallel branches. The prediction head branch received multiscale feature maps from FPN and predicted the classification information, the bounding box information and mask coefficients. The Protonet branch received the largest-size feature map from the backbone network and generated multiple prototype masks. Then, the prototype masks and mask coefficient are linearly combined to obtain the mask corresponding to each object. The final masks (the pixel-level shapes for objects) were obtained from the corresponding masks through cropping and threshold.

We leveraged a transfer learning [31] approach by using pre-trained Microsoft (MS) common objects in context (COCO) [32] weights, and then fine-tuned on a data set of fluorescent nematode images that we manually labelled. The ground truth masks are created from raw images using a python tool (Labelme, CSAIL, Cambridge, MA, USA) that allows the user to draw around each neuron; 3000 images of nematodes without N-GQDs-treatment were used for training and validation, while 200 images of each group were used for testing. The ratio among the training and validation sets was 9:1. It is worth mentioning that random combinations of left-right and up-down flips, 90 rotations, and affine shearing were utilized to augment the training set inline. Finally, to assess segmentation performance, we resorted to precision and recall, described as:

$$Precision=\frac{True\;Positive}{True\;Positive+False\;Positive}$$

$$Recall=\frac{True\;Positive}{True\;Positive+False\;Negative}$$

where true positives indicate correctly identified neurons, false positives present non-neuron objects incorrectly identified as neurons, and false negatives are neurons incorrectly identified as non-neuron objects. After hyperparameter optimization, the proposed YOLACT-based fluorescent nematode image achieved 92% in precision and 90% in recall for the entire testing set (Figure 5b). In addition, we used the Jaccard index (i.e., intersection over union) of each neuron in the testing set. The average Jaccard index for all neurons was 0.83 (std. dev = 0.12). It seems like the proposed YOLACT-based approach obtains consistent unbiased segmentation with high accuracy. Finally, the final masks obtained were used to further calculate the average number of neurons, average of mean size of neuron bodies and average fluorescence intensity in both dopaminergic neurons and glutamatergic neurons.

### 2.4. N-GQDs Induced Neurodegeneration of Neurons

**Figure 6:**
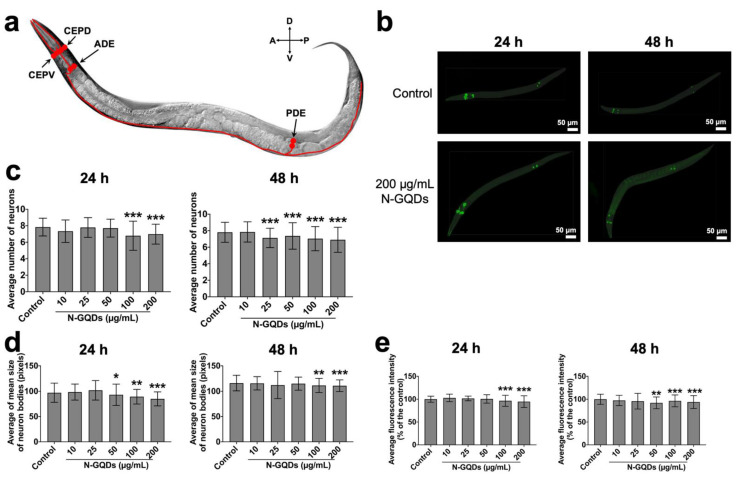
The quantitative analysis of morphological changes in dopaminergic neurons in transgenic strain of BZ555 *C. elegans* by using YOLACT algorithm. (**a**) A schematic diagram of arrangement of the four dopaminergic neuron pairs. (**b**) Representative fluorescence images of nematodes treated with saline and 200 µg/mL N-GQDs. Green boxes indicated neurons. The average number of neurons (**c**), the average of mean size of neuron bodies (pixels) (**d**), and average fluorescence intensity (% of the control) (**e**) in nematodes. L4-larvae of BZ555 and L1-larvae of BZ555 were treated with 0, 10, 25, 50, 100 and 200 µg/mL N-GQDs for 24 h without food and 48 h with food, respectively (*n* = 300). Data are shown as mean + SD of three independent experiments. The one-way ANOVA followed by the Dunnett’s *t*-test were used to determine statistical significance (* *p* < 0.05, ** *p* < 0.01, *** *p* < 0.001 vs. the control).

The location of dopaminergic neurons in *C. elegans* include four left-right pairs of neurons: one pair of cephalic sensilla-dorsal neurons (CEPD), one pair of cephalic sensilla-ventral neurons (CEPV) and one pair of class E anterior deirid neurons (ADE) in the anterior part of the nematode, and one pair of class E posterior deirid neurons (PDE) in the posterior part of the nematode (Figure 6a) [33]. When nematodes were exposed to nanomaterials, their neurons underwent morphological decline [16,34] Firstly, the morphological changes in dopaminergic neurons were quantitatively analyzed using the deep learning method. Metrics included average number of neurons, average of mean size of neuron bodies and average fluorescence intensity indicate the loss of soma, shrunken soma, and neurodegeneration of neurons because of their potential biological significance in the neurotoxicological study.

When nematodes were treated with N-GQDs at a series of dosages for 24 h or 48 h, 300 randomly selected worms of each group were analyzed using the YOLACT algorithm (Figure 6b). The number, size and fluorescence intensity of dopaminergic neurons in *C. elegans* exposed to N-GQDs at dosage above 100 µg/mL for 24 h all significantly decreased when compared to the control, while similar results were observed in nematodes exposed to N-GQDs for 48 h (Figure 6c,e), which indicated the exposure of N-GQDs with high doses could cause loss, shrinking and neurodegeneration of soma.

**Figure 7:**
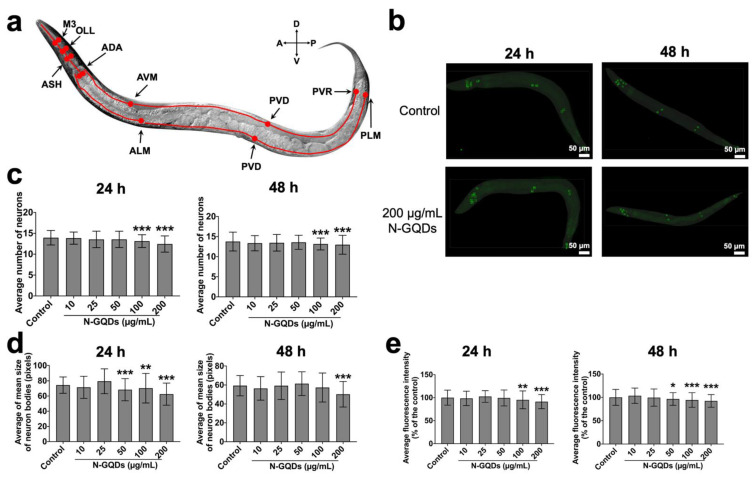
The quantitative analysis of morphological changes in glutamatergic neurons in transgenic strain of DA1240 *C. elegans* by using YOLACT algorithm. (**a**) A schematic diagram of arrangement of representative 14 glutamatergic neurons, where four neuron pairs in the head, two neuron pairs in the body and one neuron pair in the tail. (**b**) Representative fluorescence images of nematodes treated with saline and 200 µg/mL N-GQDs. Green boxes indicated neurons. The average number of neurons (**c**), the average of mean size of neuron bodies (pixels) (**d**), and average fluorescence intensity (% of the control) (**e**) in nematodes. L4-larvae of DA1240 and L1-larvae of DA1240 were treated with 0, 10, 25, 50, 100 and 200 µg/mL N-GQDs for 24 h without food and 48 h with food, respectively (*n* = 300). Data are shown as mean + SD of three independent experiments. The one-way ANOVA followed by the Dunnett’s *t*-test were used to determine statistical significance (* *p* < 0.05, ** *p* < 0.01, *** *p* < 0.001 vs. the control).

According to available studies, the number of glutamatergic neurons is large (30~40% of all neurons are thought to be glutamatergic.) so has not yet been completely identified [35,36]. Therefore, in the DA1240 transgenic strain, an easily identified 14 neurons were used to undertake the deep learning analysis, including four pairs (M3, OLL, ASH and ADA) in the nerve ring, as well as one pair (PVD) and four individuals (AVM, ALM, PVR, PLM) in the body (Figure 7a) [36]. Meantime, 300 images in each group were randomly selected to undertake the quantitative analysis by means of a trained neural network (YOLACT algorithm) (Figure 7b). The results suggested that 100 and 200 µg/mL N-GQDs exposure for 24 h or 48 h could cause glutamatergic neuron loss, shrinking and neurodegeneration in *C. elegans* as well (Figure 7c,e).

## 3. Discussion

N-GQDs have been considered as a desirable type of nanomaterial in many fields due to their excellent properties, but their rapidly growing application increases the risk to the environment and public. Recently, although the nervous system is one of the primary targets of N-GQDs, only a few analyses have been conducted on neurological effects in living animals. So far, *C. elegans* has been a reliable animal model with which to undertake ecotoxicity tests of nanomaterials, and it is sensitive to environmental neurotoxicants [14,37]. Furthermore, the nervous system of *C. elegans* are structurally and functionally conserved with vertebrate, which means the findings in *C. elegans* can be extrapolated to higher organisms [38,39]. In this study, *C. elegans* was used to assess the adverse effects of N-GQDs in the nervous system, especially the construction of neurons with the assistance of a deep learning algorithm.

The fluorescence effects of N-GQDs and optical transparency of wild-type N2 *C. elegans* without autofluorescence rendered it feasible to determine the permeable effects of N-GQDs in living bodies. The findings suggested the accumulation of N-GQDs in the whole body of the nematode, mainly in the intestine after acute exposure (24 h), while mainly in the head after sub-chronic exposure (48 h), and could result in several adverse effects in *C. elegans*. Alternations of neurons at the cellular or functional level that are dramatically affected by small variations in the environment, like nanomaterials, can profoundly change the basal behaviors of animals [14,25]. Therefore, behavioral assays offer researchers simple, sensitive and powerful tools to interrogate neuronal function.

Here, three well-characterized *C. elegans* behaviors, i.e., body bend, head thrash and pharyngeal pumping, were used as direct measures of the adverse effects of N-GQDs in the nervous system. Unfortunately, impairments on body bend, head thrash and pharyngeal pumping were all observed in nematodes treated with N-GQDs, especially when exposure doses were above 100 µg/mL and exposure time was 48 h, which indicated the potential neurotoxicity of N-GQDs. As we know, the nervous system of *C. elegans* contains almost all of the known neurotransmission systems that are phylogenetically conserved from nematodes to vertebrates [40,41], and the neurons in *C. elegans* could be classified by neurotransmitter phenotypes [27]. Here, the findings on expression changes of five neurotransmission-relevant genes assisted us to unveil the potential targets of N-GQDs in the nervous system. It seems the influences of N-GQDs in both dopaminergic and glutamatergic neurons might be responsible for their neurotoxicity in *C. elegans*.

Furthermore, two types of strains, BZ555 and DA1240, where dopaminergic and glutamatergic neurons are tagged as GFP respectively, were used to evaluate the adverse impacts of N-GQDs. Firstly, the modifications in behaviors were similar between wide-type nematodes and two transgenic strains, so we conclude that the observed adverse neurotoxic effects were due to N-GQDs exposure. After that, the neuronal survival and neurodegeneration were assessed through monitoring the integrity of neurons with the strains using a YOLACT model.

In a traditional way, the quantitative investigation of morphological changes in neurons is manually conducted with limited images, which undoubtedly results in some bias. The quantification with deep learning approach not only increases the efficiency when decreasing the manual bias, but also enables a large number of animal populations to be dealt with, which means this approach decreases the time required to process each image from hours to less than a minute without human bias and errors that are attributed to manual assessment, as well as facilitating high-content quantification of the subtle neurodegenerative changes in neurons that are usually unfeasible in conventional methods. No matter which method was used, indicators of measuring the morphological changes include the average number of neurons and neuron size, and fluorescence intensity in two types of neurons for deeper independent analysis.

Dopamine is a neurotransmitter that has been intensively investigated because of its prominent roles in the brain functions of many animals [42,43]. In *C. elegans*, dopamine is released from a limited number of neurons and is responsible for multiple functions, such as locomotor regulation, sensory perception, and learning, etc. [33,44,45]. Glutamate is the most broadly employed excitatory neurotransmitter in nervous systems of most vertebrate and invertebrate. Glutamatergic neurotransmission also occurs in *C. elegans* and glutamatergic phenotype of a neuron was defined by one well characterized VGLUT-encoding gene, eat-4 [36,46]. According to the data analyzed by the trained YOLACT algorithm, N-GQDs exposure could damage both dopaminergic and glutamatergic neurons when the administration dosages are over 100 µg/mL. Combining all findings, it seems that N-GQDs with high-dose exposure could affect the behavior of *C. elegans* by interfering with the morphology and function of dopaminergic and glutamatergic neurons.

In this study, the image analysis of neurons in *C. elegans* through a YOLACT model not only eliminates the human errors and bias induced by manual evaluation, but also facilitates high-content quantification of toxic changes that are unfeasible in conventional methods, which provides a deeper insight into the methods of measuring the neurotoxic process of nanomaterials in *C. elegans*.

## 4. Materials and Methods

### 4.1. Preparation of N-GQDs

The N-GQDs (Product No. XF241) used in this study was purchased from XFNANO Materials Tech Co., Ltd. (Nanjing, China) (http://www.xfnano.com, accessed on 18 May 2020). The K medium was used to prepare 1 mg/mL stock solution of N-GQDs, which were stored at 4 °C for 1 week. After that, they were ultrasound scattered for 30 min (40 kHz, 100 W) and filtered through 0.20 µm filter to be sterilized. The physicochemical characterizations of N-GQDs were evaluated before the study. High-resolution transmission electron microscope (HR-TEM) images were acquired on an electron microscope. The particle morphology was examined by atomic force microscopy (AFM). The FT-IR spectra were recorded on a Nicolet iS10 spectrometer (Thermo Fisher Scientific, Waltham, MA, USA). The absorption spectra and fluorescence spectra were measured on a UV-2550 spectrometer. The dynamic light scattering (DLS) and surface ξ-potential measurements were carried out on a Malvern Zetasizer Nano ZS instrument (Malvern Panalytical, Malvern, UK). The detailed characterization data of N-GQDs are shown in Table S2 and Figure S2.

### 4.2. Strains and Culture Conditions

Nematodes used in this study were wild-type N2 *C. elegans* and transgenic strains of BZ555 [egls1 (dat-1p::GFP)] and DA1240 [adls1240 (lin-15 (+) eat-4::sGFP) X]. The *C. elegans* strain and the *Escherichia coli* OP50 strain were originally obtained from the *Caenorhabditis* Genetics Center (CGC) (University of Minnesota, MN, USA). *C. elegans* culture and manipulation were performed using standard methods [47], which is that the *C. elegans* was cultured at 20 °C on nematode growth media (NGM) agar plates seeded with *Escherichia coli* OP50. Synchronization of nematode cultures was achieved using a bleaching buffer (5% 5 M NaOH, 12% NaClO) treatment of gravid hermaphrodites. All animal procedures were performed in strict accordance with the Guidelines for Care and Use of Laboratory Animals of Southeast University and experiments were approved by the Animal Experimental Ethics Committee of Southeast University (Nanjing, China).

### 4.3. Preparation of Plates and Exposure Conditions

The stock N-GQDs solution was ultrasound scattered for 30 min (40 kHz, 100 W), and diluted to the used concentrations (10, 25, 50, 100 and 200 µg/mL) in K medium for N-GQDs-treated groups just before experiments. The exposure dosages used here are according to the neuroscientific applied dosages of N-GQDs in several laboratory experiments in order to not only assess the biosafety of N-GQDs in bio-applications, but also evaluate the possible adverse effects of N-GQDs in a lab environment [48,49,50]. The acute exposure to N-GQDs was performed in synchronized L4-larvae for 24 h in 12-well sterile tissue culture plates at 20 °C in the absence of food (*E. coli* OP50), while the sub-chronic exposure to N-GQDs was performed from synchronized L1-larvae to young adults (approximately 48 h) in 12-well sterile tissue culture plates at 20 °C in the presence of food. After N-GQDs exposure, the nematodes were used for the following toxicity assessments.

### 4.4. Body Bends Assay

A body bend was counted as a change in the direction of the part of the nematodes corresponding to the posterior bulb of the pharynx along the Y axis, assuming the nematode is travelling along the X axis [51,52]. The nematodes were transferred to a second plate without food and scored for the number of body bends in an interval of 20 s with a manual counter. A randomly selected 30 nematodes were examined per treatment using a stereo microscope (Olympus, SZ61, Tokyo, Japan) and the tests were independently performed three times. Assessors were blind to the groups and each group was assessed by two assessors to ensure the data were accurate.

### 4.5. Head Thrash Assay

A head trash was defined as a change in the direction of bending at the mid body [39,53,54,55]. The nematodes were transferred to a second plate without food and scored for the number of head thrashes were scored for 20 s with a manual counter. A randomly selected 30 nematodes were examined per treatment using a stereo microscope (Olympus, SZ61) and the tests were independently performed three times. Assessors were blind to the groups and each group was assessed by two assessors to ensure the data were accurate.

### 4.6. Pharyngeal Pumping Assay

The pharyngeal pumping rate represents the food intake ability of *C. elegans*. Thirty nematodes of each group were randomly selected and their pharynx pumping frequency was determined three times over a timespan of 20 s at room temperature with a manual counter [56]. The test was performed a minimum of three times. Assessors were blind to the groups and each group was assessed by two assessors to ensure the data were accurate.

### 4.7. Quantitative Real-Time Reverse-Transcription Polymerase Chain Reaction (qRT-PCR) Analysis

Equal quantities of total RNA of worms were used to undertake qRT-PCR analysis, which was carried out as previous description [17]. The qRT-PCR primers were designed by software Primer Premier based on sequences retrieved from the *C. elegans* database (www.wormbase.org, accessed on 24 June 2020) and the National Center for Biotechnology Information (NCBI) (Tables S1 and S3). The relative quantities of mRNA were normalized against the mRNA of reference gene *act-1*. Three replicates were conducted for each qRT-PCR analysis.

### 4.8. Analysis of Morphological Laternations in Dopaminergic Neurons and Glutamatergic Neurons

The transgenic strains of BZ555 and DA1240 that are fused with GFP derived by the dat-1 promoter and eat-4 promoter, were used to visualize the dopaminergic neurons and glutamatergic neurons in nematodes with a fluorescence microscope (Olympus, FSX100), respectively. The GFP reporter transgenes have been a primary tool for visualization of cellular and physiological processes in *C. elegans* for decades and the florescence intensity is usually stable [57]. In order to avoid the influence from GFP quenching, the fluorescent images were photographed on the same day in the same fluorescent microscope with fixed exposure parameters to avoid the possible effects of variance of the light source and microscope. Meanwhile, worms of each group were visualized on prepared glass slides, and five pictures only were taken of each slide to avoid fluorescent quenching caused by the laser of fluorescent microscope. The average number of neurons, average of mean size of neuron bodies and average fluorescence intensity in both dopaminergic neurons and glutamatergic neurons were quantitatively assessed by the below YOLACT model. A randomly selected 50 nematodes were examined for each group. Three replicates were performed independently.

### 4.9. YOLACT Algorithm

YOLACT [58,59] is a representative one-stage instance segmentation model based on CNNs, which is more efficient compared with the two-stage model, e.g., Mask R-CNN [60]. Similar to Mask R-CNN, YOLACT also add a mask branch to the existing one-stage detector to achieve instance segmentation, however, it does not introduce localization steps. Firstly, YOLACT uses two CNN structures called backbone and feature pyramid network (FPN) [61] to automatically extract hierarchical sets of image features directly from input images without requiring manual feature engineering. Then, the extracted features are transmitted into two parallel branches: the Protonet branch generates a series of prototype masks which are independent of a single instance, while the prediction head branch predicts the classification information, position information, and mask coefficients. Finally, the output results of these two branches are combined to get the final prediction mask.

### 4.10. Data Analysis

All data were displayed as the mean ± standard deviation (SD). Statistical analysis was performed using Graphpad Prism 6.0 Software (GraphPad Software, San Diego, CA, USA). One-way analysis of variance (ANOVA) was used to determine the statistical significance between the control and exposed groups followed with the Tukey LSD (least significant difference) *post hoc* test to determine the significance of differences between groups. Probability levels of <0.05, <0.01 and <0.001 were considered statistically significant.

## 5. Conclusions

In this study, N-GQDs could be accumulated in the body of *C. elegans* in a time-dependent manner. The exposure of N-GQDs damaged the normal behaviors of nematodes in a dose-dependent manner, especially when the administration dosage of neurons was above 100 µg/mL. With the assistance of a deep learning approach, parameters achieved from a trained YOLACT model offered some descriptive measures that facilitated visualizing the morphological changes of neurons, including number and size of neurons and fluorescence intensity. The findings indicated that damage to dopaminergic and glutamatergic neurons may participate in the neurotoxic effects caused by N-GQDs in *C. elegans*.

## Data Availability

The data presented in this study are available on request from the corresponding author.

## Supplementary Materials

The following are available online at https://www.mdpi.com/article/10.3390/nano11123314/s1. Figure S1: The characterization of N-GQDs, Figure S2: Alterations in the expression of genes dat-1, eat-4, unc-47, unc-17 and tph-1 that are relevant to different types of neurons in *C. elegans*, Table S1: The summary of physicochemical characteristics of N-GQDs, Table S2: Designed qRT-PCR primers of genes, Table S3: Description and function of genes.
