# Supplementary material for: A Deep Learning Analysis Reveals Nitrogen-Doped Graphene Quantum Dots Damage Neurons of Nematode Caenorhabditis elegans
Source: Nanomaterials (Basel). 2021 Dec 7;11(12):3314. doi: 10.3390/nano11123314 (PMC8703693; doi:10.3390/nano11123314)
Supplement: Supplementary file 1 [file nanomaterials-11-03314-s001.zip › nanomaterials-1420715-supplementary.pdf]

nanomaterials

MDPI

Supporting Information

A Deep Learning Analysis Reveals Nitrogen-Doped Graphene Quantum Dots Damage Neurons of Nematode *Caenorhabditis elegans*

Hongsheng Xu[1], Xinyu Wang[2], Xiaomeng Zhang[2], Jin Cheng[2], Jixiang Zhang[2], Min Chen[2] and Tianshu Wu[2,*]

[1] College of Energy and Electrical Engineering, Hohai University, Nanjing 210098
[2] Key Laboratory of Environmental Medicine and Engineering, Ministry of Education; School of Public Health, Southeast University, Nanjing 210009, China
* Correspondence: Author to whom correspondence should be addressed. E-Mail: ninatswu@126.com/ninatswu@seu.edu.cn

Table S1. Description and function of genes.

Gene.	Description	Function
dat-1	DopAmine Transporter-1	Dopamine-transporter
eat-4	EATing: abnormal pharyngeal pumping-4	Vesicular glutamate transporter
unc-47	UNCoordinated-47	Vesicular GABA transporter
unc-17	UNCoordinated-17	Vesicular acetylcholine transporter
tph-1	TryPtophan Hydroxylase-1	Serotonin synthesis

Table S2. The summary of physicochemical characteristics of N-GQDs.

QDs	Mean size by TEM (nm)	Thickness (nm)	Mean size by DLS in K medium (nm)	ξ-potential in K medium (mV)	Excitation peak in K medium (nm)	Emission peak in K medium (nm)	PLQY (%)
N-GQDs	~3	0.5~3	5.0	-10.4	380	480	~20

Table S3. Designed qRT-PCR primers of genes.

Gene name	Designed qRT-PCR primers	
	Forward	Backward
dat-1	GGAGTGACTCTACCCGGATG	CTCCGAATCCTGGCCCTAAT
eat-4	GTGGTCCTTATGGGTGGACA	TGAATGCAGCTTCTCCTCCA
unc-47	CCCACCTCATGACCGCTTGG	AGCACCCCTGTCCAGTAGCA
unc-17	TCACAACCTGGATGTCCGAA	TCCGAGAACGTGTGGAAAGA
tph-1	TGCAACATGCCGTTGAGGGT	CGGCGAGCAGGTTGATGTCT
act-1	GCTGGACGTGATCTTACTGATTACC	GTAGCAGAG CTTCTCCTTGATGTC

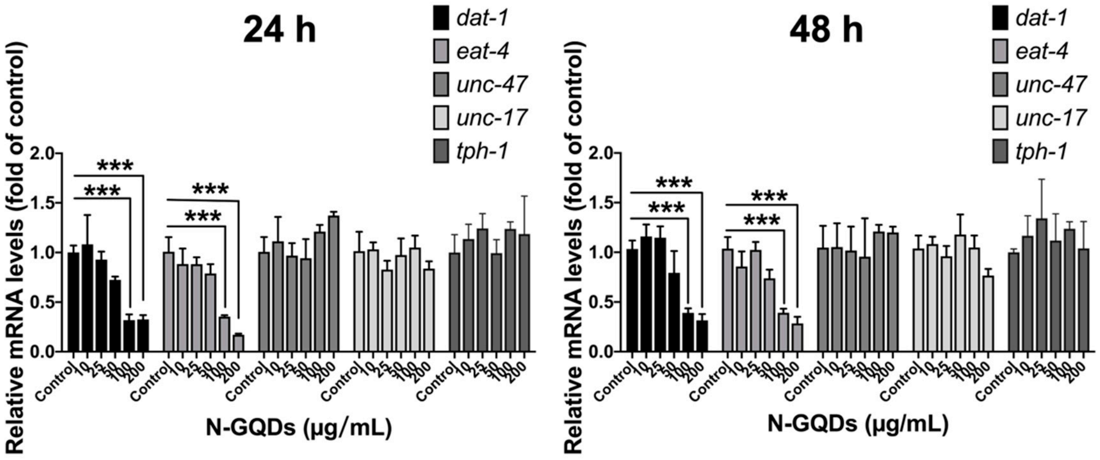

Figure S1. Alterations in the expression of genes dat-1, eat-4, unc-47, unc-17 and tph-1 that are relevant to different types of neurons in C. elegans. L4-larvae of N2 and L1-larvae of N2 were treated with 0, 10, 25, 50, 100 and 200 μg/mL N-GQDs for 24 h without food and 48 h with food, respectively (n=30). Data are showed as mean+SD of three independent experiments. The one-way ANOVA followed by the Dunnett's t test were used to determine statistical significance (***P<0.001 vs the control).

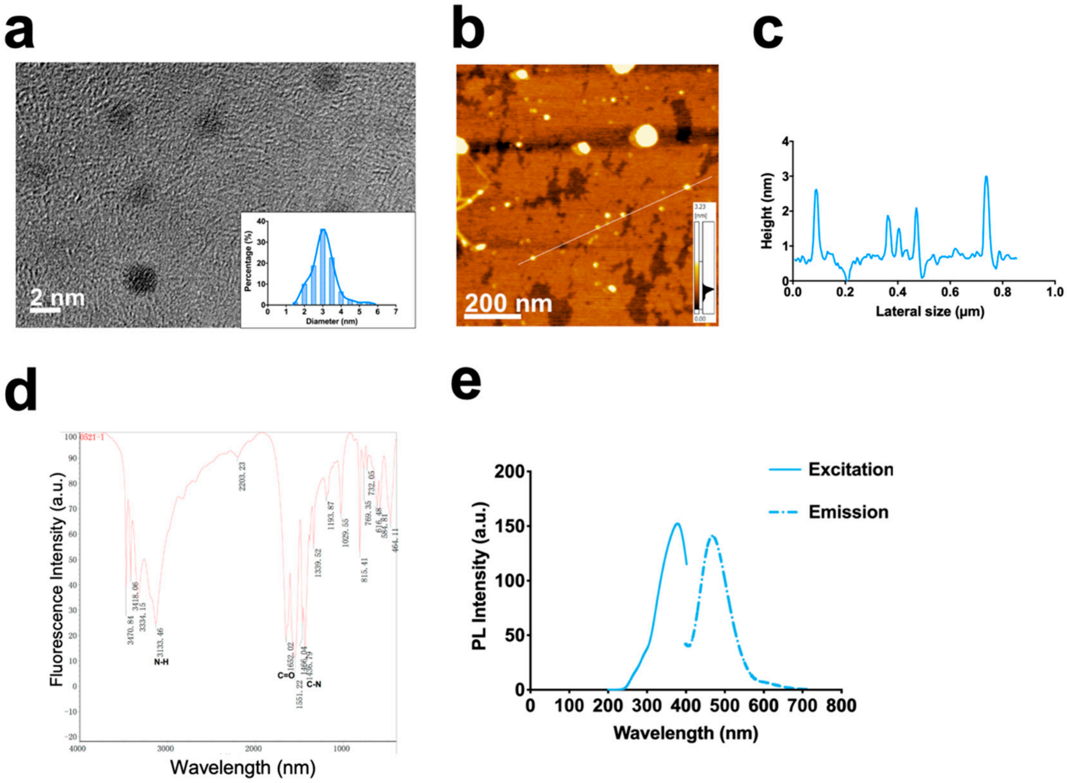

Figure S2. The characterization of N-GQDs. (a) The TEM images and size distribution of N-GQDs; (b, c) The AFM topography and height distribution of N-GQDs; (d) The FT-IR spectrum of N-GQDs; (e) The excitation and emission spectra of N-GQDs in K medium at λem = 420 nm and 480 nm, or λex = 350 nm and 380 nm.
